# The complete mitochondrial genome of *Sorubim Lima* (Siluriformes, Pimelodidae)

**DOI:** 10.1080/23802359.2019.1678420

**Published:** 2019-10-18

**Authors:** Fang Ren, Xiuhui Ma

**Affiliations:** a School of Animal Science, Guizhou University, Guiyang, Guizhou Province, China;; b Key Laboratory of Animal Genetics, Breeding and Reproduction in The Plateau Mountainous Region, Ministry of Education, Guizhou University, Guiyang, Guizhou Province, China

**Keywords:** Mitochondrial genome, *Sorubim lima*, next-generation sequencing

## Abstract

The complete mitochondrial DNA genome of *Sorubim lima* was first reported by next-generation sequencing method. The entire length of mitochondrial genome is 16,539 bp and the nucleotide composition was made up of 32.1% A, 24.9% T, 28.0% C, and 15.0% G, indicating an A + T (57.0%)-rich feature. With the exception of 8 tRNA genes and NADH6, mitochondrial genes are encoded on the heavy strand, which was similar to that in other vertebrates. The results showed that the species of Pimelodidae were gathered in the same branch. The phylogenies indicate monophyly of the genus *Pseudoplatystoma*, *Pimelodus* and *Sorubim*.

The Pimelodidae, commonly known as the long-whiskered catfishes, belongs to Siluriformes, which are distributed from South America and Panama north to southernmost Mexico (Nelson 2006). There are 32 genera and 113 valid species in this family in fishbase, but only 7 complete mt DNA sequences are available (Sato et al. 2018). Specimens of *Sorubim lima* were collected from Huadiwan Flowers & Birds market in Guangzhou, China (N: 23°05’13.76''; E: 113°13'34.52''). Voucher specimens (gzu20170831) were preserved in 100% ethanol and deposited at zoological museum of the School of Animal Science, Guizhou University. In the present study, the complete mitochondrial DNA genome of *S. lima* was first reported. The complete mitochondrial sequence of *S. lima* had been deposited in the GenBank with an accession number MN242829. The entire length of mitochondrial genome is 16,539 bp and the nucleotide composition was made up of 32.1% A, 24.9% T, 28.0% C, and 15.0% G, respectively, indicating an A + T (57.0%)-rich feature. The mitochondrial sequence was annotated with the web-site based tool DOGMA (Wyman et al. 2004) and 22 tRNA genes were scanned by tRNAscan-SE (Lowe and Chan 2016). The mitogenome includes 22 transfer RNA genes, 2 ribosomal RNA genes, 13 protein-coding genes and one non-coding control region (D-loop). A total of 71 bp intergenic nucleotides ranging from 1 to 30 bp were identified in the whole genome. Except for overlapped between NADH5 and NADH6 encoded on opposite strands, some overlaps were also appeared on 13 protein-coding genes encoded on the same strand. The similar phenomena have already shown in most vertebrates (e.g. Gu et al. 2007; Vittas et al. 2011).

The phylogenetic relationship of *S. lima* and other Pimelodidae species was inferred using the neighbor-joining and maximum likelihood method in MEGA 7.0 (Kumar et al. 2016). The results showed that the species of Pimelodidae were gathered in the same branch. The phylogenies indicate monophyly of the genus *Pseudoplatystoma*, *Pimelodus* and *Sorubim* with very high support values (BP = 100, Figure 1). The *Pseudoplatystoma* genera was placed with *Sorubim* to form a sister group to *Pimelodus* in the phylogenetic tree. *S. lima* and *Sorubim cuspicaudus* were sister group (Figure 1). This report could enrich the mitogenome resource of *S. lima* and it also seems to be useful for evolutionary and conservation studies on Pimelodidae and Siluriformes fish species

**Figure 1.**
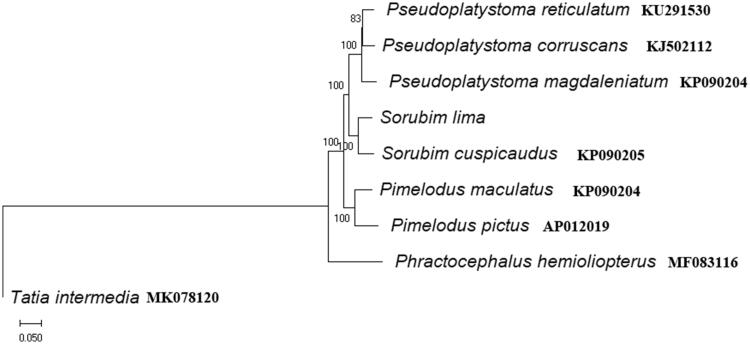
Neighbor-joining phylogenetic tree of the *S. lima* and other species based on the complete mitochondrial genome. Numbers on nodes indicate bootstrap support value, based on 1000 replicates.
